# Medical Women in England

**Published:** 1891-03-14

**Authors:** 


### The Hospital

A Weekly Journal of

**Science, Medicine, Nursing, and Philanthropy.**

*For Hospitals, Asylums, Infirmaries, Dispensaries, Medical Practitioners, Students, Nurses, and the Charitable Public.*

Vol. IX.—No. 233.] [March 14, 1891.

### Medical Women in England.

Some of the facts brought before the Lords' Committee last week by Mrs. Garret Anderson, M.D., are such as should give great dissatisfaction to men, and more particularly to cultivated and reasoning medical men. We are surely entitled to expect reason at the hands of reasonable creatures, and liberal judgments from the minds of those who have been liberally educated. Women have claimed the right to work freely at any calling into which they have been able to effect an entrance. Their right has now been acknowledged by all rational persons. It is felt that on purely national grounds no argument can stand which opposes the claim of woman to do anything that she finds herself able to do. She has fought her battle, and she has won it.

But though the battle has been fought on the field of reason, and the victory has gone to the just cause of women, they can by no means, as yet, gain possession of the proper fruits of victory. The medical profession is open to them in the abstract; but when they try to enter it they find many of its gateways obstinately locked in their faces. The London College of Physicians, and the London College of Surgeons, still refuse to receive women, says Mrs. Garret Anderson. The London University, on the other hand, flings wide its gates. One cannot help wondering what the individual men who constitute the governing body of the College of Physicians or the College of Surgeons think of themselves in this matter. If one of them could be made to stand by himself and to give a true and full account of his motives in standing up against the weaker sex with a professional revolver in his hand to drive them back from the approaches to his own gateway—would he have the grace and the manliness to be ashamed of the account when he saw it written down?

No man ever takes a definite line of action without being influenced by a motive of some kind. What is the motive of each individual member of the governing body of the College of Physicians or the College of Surgeons? Is it, in so many words, the desire to limit professional competition? If it be not that, is it that the members of the governing bodies have not the intellectual capacity to see that in reason a woman has precisely the same right to enter upon any kind of work she can fit herself for as a man has? It must be one or other of these reasons that act as motives upon the individual members of the governing bodies of the colleges. But either of these states of mind is a mental condition of which any man of education ought to be ashamed. It is quite certain, indeed, that almost any particular member of either of these governing bodies would, if placed by himself and confronted with his own opinions, have the grace to be ashamed of them. But when it comes to a question of voting and definite action he goes with the crowd, and silences his conscience by putting the responsibility upon his confrères.

The methods described are such as might be practised by uninstructed trades' unionists without striking one as being either very peculiar or very unworthy. Though the methods would be unworthy in themselves, yet we do not expect from large masses of uneducated men a very clear appreciation of reasonableness in the abstract or a high degree of chivalrous unselfishness in actual behaviour. But of educated men we expect the fruits of education; and at the hands of gentlemen we look for chivalry. Now, what are the fruits of education? One of the first is the power of abstract reasoning, and of seeing what is just and right in practical conduct. More especially do we expect that this fruit of education will appear when men take part together in important public acts. Those who legislate for kingdoms should be statesmen; free from prejudice, and uninfluenced by self-interest. Those who legislate for colleges should, in all respects, conform to this same standard. No one denies this in the abstract. Why then, in the name of justice, in the name of conscience, in the name of intelligence and plain sense, do we do that in the concrete which we should be ashamed to do in the abstract? Why does any man join with a multitude to do a thing of which he would be ashamed if he were asked to do it alone?

It appears from Mrs. Garrett Anderson's statement that many women students take the greater part of their curriculum at the Royal Free Hospital. But at that institution they can get no practice at all in the art of midwifery—the very art which they ought most to cultivate. Women, as a matter of fact, find the greatest possible difficulty in obtaining the twenty or thirty midwifery cases needed for the acquiring of a regular medical qualification. "They had twice," said Mrs. Garrett Anderson, "asked the Royal Free Hospital to establish a midwifery department, but the hospital did not appear to like it." Now, one really hardly knows what to say which shall be strong enough, and biting enough, to eat its way into the heads and consciences of the medical profession in general, and of the managers of the Royal Free Hospital in particular, on this subject. What one would like to see is at least a little manly consistency among medical men, hospital managers, and examining boards. What one does actually see is that certain almost worthless concessions are held out to women with one hand, and forcibly pulled back by the other. Those concessions, we believe, are cowardly concessions, made in deference to public opinion. They are not worth the making. It would be much more manly and honourable to refuse them. A fine old crusted Conservatism commands everybody's res pect It has lived by a certain rule, and it will die by the same rule. But a timid, hesitating, apologetic, and "vanishing ghost" policy is worthy only of contempt. If the Royal Free Hospital desires to have the credit of educating women, let it educate them properly, and let it be ashamed to do anything else.

There is a great deal of keen competition in the medical profession. That, the writer knows well, both by his own experience and by that of his professional friends. But no amount of competition, however desperate it may be, can justify a man in acting as a lion would act who had just secured a buffalo-calf, or as a dog with a solitary bone. The lion acts after his kind, and the dog after his. But the man must act after his kind. Cynical selfishness is not merely the devil's policy: it is the fool's policy as well. Women have an indefeasable right to gain their livelihood by the doing of any work for which they may make themselves competent. To try to prevent them is to practice a tyranny; a weak, foolish and base tyranny; a tyranny which will prove not even profitable to its upholders in the long run. Men should hold out the hand of friendliness and help to women; they should welcome them most eagerly to the intellectual callings. They ought to be chivalrous, and to smooth the pathway; they should carry out in public that which they profess to practise and do practise in private. Was the world made for men only? Has woman earned no right to toil? Is it not to her honour that she wishes to work for herself, and to be a burden upon no man? Medical men pursue a domestic calling; they see woman at home; they see her in the sick room; they know her capacity for managing the sick; they know the peculiar devotedness of the womanly heart. Are not they themselves the first to ask for a woman's ministrations when they are helpless and sick? Let them take the matter to the high court of reason; let them take it to the still higher tribunal of an honest and unsophisticated conscience. The stage of development at which the cultivated races have arrived demands the intellectual and practical emancipation of women. It is medical men who best understand the progress of the world from the philosophic and developmental point of view. Let them acknowledge the justness of their own theories and stand by those theories firmly and loyally in practice.

